# Sensitivity to change and prediction of global change for the Alzheimer’s Questionnaire

**DOI:** 10.1186/s13195-014-0092-z

**Published:** 2015-01-08

**Authors:** Michael Malek-Ahmadi, Kewei Chen, Kathryn Davis, Christine M Belden, Jessica Powell, Sandra A Jacobson, Marwan N Sabbagh

**Affiliations:** The Cleo Roberts Center for Clinical Research, Banner Sun Health Research Institute, 10515 West Santa Fe Drive, Sun City, AZ 85351 USA; Banner Alzheimer’s Institute, Phoenix, AZ 85006 USA

## Abstract

**Introduction:**

Longitudinal assessment of cognitive decline in amnestic mild cognitive impairment (aMCI) and Alzheimer’s disease (AD) often involves the use of both informant-based and objective cognitive assessments. As efforts have focused on identifying individuals in pre-clinical stages, instruments that are sensitive to subtle cognitive changes are needed. The Alzheimer’s Questionnaire (AQ) has demonstrated high sensitivity and specificity in identifying aMCI and AD; however its ability to measure longitudinal change has not been assessed. The aims of this study are to assess the sensitivity to change of the AQ and to determine whether the AQ predicts change in global cognition and function in cognitively normal (CN), aMCI, and AD subjects.

**Methods:**

Data from 202 individuals participating in a brain and body donation program were utilized for this study (101 CN, 62 aMCI, 39 AD). AD and aMCI individuals were matched on age, education, and gender to CN individuals. Sensitivity to change of the AQ was assessed in addition to the AQ’s ability to predict change in global cognition and function. The Mini Mental State Exam (MMSE) and Functional Activities Questionnaire (FAQ) were used as gold standard comparisons of cognition and function. Sample size calculations for a 25% treatment effect were also carried out for all three groups.

**Results:**

The AQ demonstrated small sensitivity to change in the aMCI and CN groups (*d* = 0.33, *d* = 0.23, respectively) and moderate sensitivity to change in the AD group (*d* = 0.43). The AQ was associated with increases in the Clinical Dementia Rating Global Score (OR = 1.20 (1.09, 1.32), *P* <0.001). Sample size calculations found that the AQ would require substantially fewer subjects than the MMSE given a 25% treatment effect.

**Conclusions:**

Although the AQ demonstrated small sensitivity to change in aMCI and CN individuals in terms of effect size, the AQ may be superior to objective cognitive tests in terms of required sample size for a clinical trial. As clinicians and researchers continue to identify and treat individuals in earlier stages of AD, there is a need for instruments that are sensitive to cognitive changes in these earlier stages.

## Introduction

Longitudinal assessment of cognitive decline in amnestic mild cognitive impairment (aMCI) and Alzheimer’s disease (AD) often involves the use of both informant-based and patient-based assessments to measure the degree of change in cognition and function [1,2]. In both clinical and research settings, the two methods are often used in conjunction in order to glean a more accurate picture of an individual’s current cognitive status relative to baseline or other prior time points. A major issue that both clinicians and researchers grapple with is the degree to which a particular instrument is sensitive to change over time. For clinicians, determining the significance of change from one time to the next has implications for decisions regarding treatment and resource use (that is, assisted living, in-home care, and so on.). Clinicians may also benefit from instruments that are sensitive to change over time in order to satisfy the Affordable Care Act’s cognitive screening requirement for Medicare recipients. For researchers and clinical trialists, the issue of sensitivity to change for a particular instrument has significant ramifications for whether or not a meaningful treatment effect will be detected between placebo and treatment groups.

The need to identify individuals as early as possible in the AD disease process has prompted researchers to begin conducting studies with individuals who are classified as having pre-symptomatic AD. Although no formal diagnostic criteria currently exist for this classification, it is used to classify individuals whose biological markers are consistent with the pathological presence of AD, but who are cognitively normal and are considered to be at risk for eventually developing clinical AD. An interesting study by Riley *et al*. [3] compared cognitively normal individuals who, at autopsy, met National Institute on Aging (NIA)-Reagan criteria for no- and low-likelihood of AD with cognitively normal individuals who met criteria for intermediate- and high-likelihood of AD. This study found that the intermediate- and high-likelihood groups had a steeper rate of decline on several cognitive measures across several domains, although all individuals in the study were within normal limits on cognitive testing. Riley *et al*. [3] suggest that rates of longitudinal cognitive decline may be informative in identifying individuals with pre-symptomatic AD, even when cognitive testing falls within normal limits. Gavett *et al*. [4] found that informant-reported cognitive symptoms on the Informant Questionnaire on Cognitive Decline in the Elderly (IQCODE) correlated well with longitudinal neuropsychological performance and that informant-reported changes in cognition were a robust predictor of cognitive decline in a high-functioning, cognitively normal group. Both of these studies demonstrate that cognitive decline in cognitively normal individuals can be reliably detected and may be used to predict subsequent development of clinical AD.

The Alzheimer’s Questionnaire (AQ) was originally introduced in 2010 [5] and has been validated as an accurate informant-based measure of cognition and function for both aMCI and AD [5-7]. The AQ also correlates well with established measures of cognition and global function [8]. Although the AQ has demonstrated its validity in cross-sectional studies, its ability to accurately measure change in cognition over time has not been assessed. Instruments such as the Mini Mental State Exam (MMSE) [9] and the Functional Activities Questionnaire (FAQ) [10] are commonly used to assess changes in cognition and function in aMCI and AD. Clark *et al*. [11] report that although the MMSE may be sufficient to use as a screening instrument for cognitive impairment, its utility as an instrument to assess change over time accurately is limited by high measurement error and high variability of annual change between individuals. A recent study by Costa *et al*. [12] found that the Montreal Cognitive Assessment (MoCA) yielded small sensitivity to change in prodromal AD and moderate sensitivity to change in mild AD. Recent studies suggest that the FAQ is a significant predictor of conversion to AD from aMCI [13] and has also been associated with longitudinal decreases in glucose metabolism associated with aMCI and AD [14]. Rizk-Jackson *et al*. [15] found that the FAQ was able to detect functional decline in cognitively normal individuals prior to the presence of impairment on objective cognitive tests.

The first aim of this study was to assess the sensitivity to change of the AQ through the use of effect size and sample size calculations for a hypothetical placebo-controlled clinical trial. For comparison, the MMSE and FAQ were also used in order to gauge the AQ’s performance against instruments that have been more widely used. The second aim of the study was to determine how well one-year change in AQ total score predicts global change as measured by the Functional Assessment Staging Test (FAST) [16], Global Deterioration Scale (GDS) [17], and the Clinical Dementia Rating Global Score (CDR-GS) [18].

## Methods

### Study sample

Data from the two most recent annual visits for 202 individuals participating in a brain and body donation program [19] were utilized for this study. Participants in this program were recruited predominantly from the northwest region of the Phoenix, Arizona metropolitan area. Approval for the brain and body donation program was granted by the Banner Health Institutional Review Board and informed consent was obtained from all individuals prior to enrolling in the program. The sample for this study ranged in age from 57 to 97 years with a mean of 81.70 ± 7.25 and had a mean education level of 14.74 ± 2.54 years and included 95 women and 107 men.

Of the 202 individuals, 101 were classified as cognitively normal (CN), 62 were classified as amnestic mild cognitive impairment (aMCI), and 39 were classified as Alzheimer’s disease (AD) at the first visit. Each aMCI and AD individual was matched on age, education, and gender to a CN individual, without replacement. When an exact match could not be found, a tolerance of ± 2 years was used for age and education in order to obtain an appropriate match. Both single and multiple domain aMCI cases were categorized as aMCI and both possible and probable AD were categorized as AD. The AD cases met National Institute of Neurological and Communicable Disorders and Stroke – Alzheimer’s Disease and Related Disorders Association (NINCDS-ADRDA) criteria [20] for a clinical diagnosis of probable or possible Alzheimer’s disease. aMCI cases were diagnosed as such based on Petersen criteria [21]. The CN cases were defined as having no limitations of activities of daily living by informant report and were within normal limits on neuropsychological testing.

Consensus diagnosis with a neurologist, geriatric psychiatrist and neuropsychologist was used to determine the clinical status of each individual. Consensus diagnoses were made based on neuropsychological testing results, neurological and physical exam, and interviews with an informant that assessed global cognitive status, functional status, and mood and behavioral status.

### Instruments

AQ [5,6] – A 21-item, informant-based dementia assessment designed for ease of use in a primary care setting. AQ items are divided into five domains including Memory, Orientation, Functional Ability, Visuospatial Ability, and Language. Items are posed in a yes/no format with the sum of ‘yes’ items equaling the total AQ score (0-27). Six items known to be predictive of a clinical AD diagnosis are weighted more heavily in the total score by each being worth two points rather than one.

FAQ [11] – An informant-based measure of instrumental activities of daily living (IADLs) which scores 10 items on a 0 to 3 scale, with higher scores corresponding to greater impairment.

MMSE [9] – A brief, 30-item cognitive screening instrument that includes items on Orientation, Memory, Attention, Language and Visuospatial functions.

FAST [16] – A dementia staging instrument that classifies individuals as Normal Aging, Possible Mild Cognitive Impairment, Mild Cognitive Impairment, Mild Dementia, Moderate Dementia, Moderately Severe Dementia and Severe Dementia using a 1 to 7 scale where higher ratings indicate greater severity.

GDS [17] – A dementia staging instrument divided into seven different stages with increasing impairment corresponding with higher stages (No Cognitive Decline, Age-Associated Memory Impairment, Mild Cognitive Impairment, Mild Dementia, Moderate Dementia, Moderately Severe Dementia, Severe Dementia).

CDR [18] –A semi-structured, informant-based clinical staging instrument that characterizes six domains of cognitive and functional performance: Memory, Orientation, Judgment and Problem Solving, Community Affairs, Home and Hobbies, and Personal Care. The CDR provides a global score which is a composite score based on an algorithm that gives different weights to the scores for each of the domains. The global score (GS) is used to grade the severity of dementia and is measured using 0, 0.5, 1, 2, and 3 to denote no impairment, very mild dementia, mild dementia, moderate dementia, and severe dementia, respectively.

### Statistical analysis

The Shapiro-Wilk test was performed on the data to determine the normality of distribution for the continuous variables. Non-parametric tests for group comparisons and correlations were used, as the data for all continuous variables were not normally distributed. The Kruskall-Wallis test was used to verify that the three groups were not significantly different in terms of age and education. Chi-square analysis was used to examine the distribution of men and women among the three groups.

The analyses investigating the sensitivity to change utilized a method similar to that of Costa *et al*. [11]. Middel and von Sonderen [22,23] described these methods and their rationale in detail. The sensitivity to change assessment was completed through the calculation of an effect size (ES) to quantify the magnitude of change. Since this study used a correlated design, the pooled standard deviation was used to calculate the ES which was taken from the individual standard deviation values for Year 1 and Year 2 for each measure (pooled standard deviation = √(((Year 1 sd)^2^ + (Year 2 sd)^2^)/2); (ES = mean change score/pooled standard deviation)). The final effect size measure, *d*, included a correction for reliability (*d* = ES/√2(1-*r*)) where *r* is the correlation between the scores at Year 1 and Year 2. The interpretation for *d* utilized the following scheme proposed by Cohen [24]: <0.20 = trivial change; 0.20 to 0.50 = small change; 0.50 to 0.80 = moderate change; ≥0.80 = large change.

In order to provide a more practical interpretation of the sensitivity to change, a series of sample size calculations were carried out to show how many individuals would be needed for a clinical trial using a particular measure as its outcome. The sample size calculations assumed a 25% treatment effect on the mean change score for each measure at 80% power with a two-tailed significance level of 0.05 for a randomized clinical trial with a treatment arm and a placebo arm. These parameters were used as they have been utilized by several previous studies [25] and have also been used to estimate sample sizes for pre-dementia trials using data from the Alzheimer’s Disease Neuroimaging Initiative [26]. Sample size calculations were carried out using G*Power 3 [27]. The reported sample sizes are the number per arm. For each of the clinical groups, varying trial lengths were used in the sample size calculations: AD = two years, MCI = three years, CN = five years.

To further examine the ability of each instrument to detect clinically significant change, a reliable change index (RCI) was calculated for each instrument. For this study, two different RCI methods were utilized as the AQ and FAQ are informant-based assessments and the MMSE is an objective performance-based assessment. For the AQ and FAQ, RCI calculations that corrected for inter-test reliability were used [28] while the MMSE RCI calculation utilized a method that corrects for both inter-test reliability and practice effects [29]. The most common convention for interpreting RCI scores is that scores that are ≥ ± 1.645 are interpreted as demonstrating clinically significant change [30]. This was used to obtain 90% confidence intervals for estimates of clinically significant change for each instrument from Year 1 to Year 2. In this study, we report the percent of individuals who demonstrated annual score changes outside the range of the 90% confidence interval for each instrument.

An additional set of analyses were carried out to determine the extent to which the mean change scores of the AQ, FAQ and MMSE predicted global change as measured by increases in FAST, GDS and CDR-GS values. The CN, AD and aMCI groups were analyzed separately. An analysis with the entire sample was also carried out. All individuals were dichotomized based on whether their individual FAST, GDS and CDR-GS values increased from Year 1 to Year 2 (1 = increase, 0 = no increase) as increases on these scales represent clinically meaningful changes in disease severity. Logistic regression analyses were used to assess the predictive value of the AQ, FAQ and MMSE change scores on increases in FAST, GDS or CDR-GS. A False Discovery Rate (FDR) significance level of 0.006 was used to correct for multiple comparisons within each of the groups.

Spearman correlation analyses were carried out to assess the linear associations between AQ, FAQ and MMSE scores with the FAST, GDS and CDR-GS for Year 1 and Year 2 separately. Spearman correlation was also used to assess the associations between the change scores on the AQ, FAQ, MMSE and MoCA. The correlations used as the measures of test-retest reliability are also Spearman values. Statistical analyses were carried out using Systat 12.0 (Systat, Inc., San Jose, CA, USA).

## Results

Demographic characteristics of the entire study sample and each clinical group are shown in Table 1. The three clinical groups did not differ in terms of age or years of education and there was no significant difference in gender composition among the three groups.

**Table 1:** **Demographic characteristics**

**Characteristic**	**CN**	**MCI**	**AD**	**Total**	***P*** **-value**
Number	101	62	39	202	-----
Age	81.76 ± 7.23	81.57 ± 7.59	81.82 ± 6.92	81.71 ± 7.25	0.99
Education	14.69 ± 2.50	15.18 ± 2.56	14.15 ± 2.55	14.74 ± 2.54	0.12
Gender (M/F)	53/48	33/29	21/18	107/95	0.99

Mean ± standard deviation. AD, Alzheimer’s disease; CN, cognitively normal; MCI, mild cognitive impairment; M/F, male/female.

The results from the sensitivity to change analysis are shown in Table 2. In the aMCI group the AQ, FAQ and MMSE all demonstrated small sensitivity to change in terms of their respective *d* values (0.33, 0.35, 0.24). However, both the AQ and FAQ yielded required sample sizes that were less than half of the sample size required by the MMSE.

**Table 2:** **Sensitivity to change comparison for the AQ, FAQ, and MMSE in amnestic mild cognitive impairment, Alzheimer’s disease, and cognitively normal cases**

**Group**	**Instrument**	**Mean change**	**SD of mean change**	**95% CI of mean change**	**Pooled SD**	**ES**	**Reliability**	***d***	**Required sample size** ^**a**^
aMCI									
	AQ	1.66	4.96	(0.40, 2.92)	6.10	0.27	0.65	0.33	251
	FAQ	2.05	5.79	(0.54, 3.56)	3.29	0.30	0.63	0.35	224
	MMSE	-0.55	2.54	(-1.19, 0.10)	2.47	0.22	0.56	0.24	597
AD									
	AQ	2.49	4.77	(0.94, 4.03)	6.75	0.37	0.64	0.43	232
	FAQ	3.59	4.91	(2.00, 5.18)	5.36	0.52	0.81	0.84	119
	MMSE	-2.13	3.35	(-3.23, -1.03)	6.34	0.34	0.79	0.52	157
CN									
	AQ	0.47	2.73	(-0.07, 1.00)	3.37	0.14	0.69	0.18	340
	FAQ	0.33	1.80	(-0.03, 0.69)	3.29	0.11	0.73	0.15	300
	MMSE	0.05	1.71	(-0.29, 0.39)	2.47	0.02	0.41	0.02	660

^a^Number of subjects per arm based on a 25% treatment effect of the mean change at 80% power with a two-tailed significance level of 0.05; sample size estimates are based on a two-year study for AD, a three-year study for aMCI, and five-year study for CN; for AQ and FAQ positive mean change scores represent increased impairment over time and negative mean change scores for MMSE also represent increased impairment over time. Effect size values are reported in absolute values and represent the magnitude of the difference for Year 2 scores subtracted from Year 1 scores. AD, Alzheimer’s disease; aMCI, amnestic mild cognitive impairment; AQ, Alzheimer’s Questionnaire; 95% CI, 95% confidence interval; CN, cognitively normal; *d*, effect size corrected for reliability; ES; effect size using pooled standard deviation; FAQ, Functional Activities Questionnaire; MMSE, Mini Mental State Exam; SD, standard deviation**.**

In the AD group, the AQ demonstrated small sensitivity to change (*d* = 0.43),; however, the FAQ showed large sensitivity to change (*d* = 0.84) and the MMSE demonstrated moderate sensitivity to change (*d* = 0.52). In terms of required sample size the FAQ yielded the lowest value (n = 119) while the AQ yielded a value that was substantially higher (n = 232). This result may be explained by the reliability values for each instrument as the FAQ had a higher reliability value (r = 0.81) than the AQ (r = 0.64). The MMSE yielded a required sample size that was between that of the AQ and FAQ (n = 157).

In the CN group all three measures demonstrated trivial sensitivity to change. However, sample size calculations demonstrated that the MMSE would require substantially more subjects than both the AQ and FAQ.

Results from the RCI score calculations are shown in Table 3. For the aMCI group, the AQ yielded a higher percentage of individuals demonstrating clinically significant change when compared to the FAQ and MMSE. For the AD group, the AQ yielded a higher percentage of individuals demonstrating clinically significant change when compared to the FAQ, but demonstrated an equivalent percentage compared to the MMSE. Table 4 shows the results of the predictive ability of AQ, FAQ and MMSE mean change scores on increases in FAST, GDS and CDR-GS values. Within each of the clinical groups, no statistically significant effects were found after adjusting for multiple comparisons. When all three groups were pooled together, the AQ and FAQ demonstrated small, but significant associations with CDR-GS increases (AQ (odds ratio (OR) = 1.20 (1.09, 1.32), *P* <0.001); FAQ (OR = 1.21 (1.11, 1.33), *P* <0.001)). The pooled analysis also yielded a small, but significant association for FAQ mean change and GDS increase (OR = 1.16 (1.06, 1.26), *P* = 0.001).

**Table 3:** **Reliable change index results based on data from cognitively normal individuals**

**Instrument**	**Reliability**	**SE**_**m**_	**SE**_**diff**_	**90% CI for RCI**	**Percent outside of 90% CI**
AQ	0.69	0.27	2.66	±4.37	aMCI = 24%; AD = 16%
FAQ	0.73	0.18	2.24	±3.67	aMCI = 17%; AD = 12%
MMSE	0.*41*	0.17	1.73	±2.84	aMCI = 17%; AD = 17%

AD, Alzheimer’s disease; AQ, Alzheimer’s Questionnaire; CI, confidence interval; FAQ, Functional Activities Questionnaire; MMSE, Mimi Mental State Exam; RCI, reliable change index; SE_diff_, standard error of the difference; SE_m_, standard error of measurement.

**Table 4:** **AQ, FAQ, and MMSE mean change as predictors of global change in mild cognitive impairment, Alzheimer’s disease, cognitively normal cases, and all groups combined**

**Group**	**Instrument**	**FAST increase**	**GDS increase**	**CDR Global score increase**
aMCI				
	AQ	1.07 (0.96, 1.20); *P* = 0.23	1.09 (0.97, 1.22); *P* = 0.15	1.09 (0.97, 1.24); *P* = 0.16
	FAQ	1.07 (0.96, 1.19); *P* = 0.25	0.97 (0.87, 1.08); *P* = 0.52	1.08 (0.96, 1.22); *P* = 0.22
	MMSE	1.02 (0.83, 1.26); *P* = 0.83	0.81 (0.64, 1.02); *P* = 0.07	0.91 (0.72, 1.15); *P* = 0.43
AD				
	AQ	1.17 (0.97, 1.29); *P* = 0.14	1.26 (1.05, 1.52); *P* = 0.01	1.16 (1.00, 1.35); *P* = 0.06
	FAQ	1.09 (0.95, 1.24); *P* = 0.22	1.17 (1.00, 1.38); *P* = 0.05	1.12 (0.97, 1.29); *P* = 0.12
	MMSE	0.93 (0.77, 1.14); *P* = 0.50	0.97 (0.79, 1.19); *P* = 0.77	0.91 (0.74, 1.11); *P* = 0.35
CN				
	AQ	1.09 (0.93, 1.28); *P* = 0.29	1.04 (0.88, 1.23); *P* = 0.67	1.26 (1.00, 1.59); *P* = 0.05
	FAQ	0.91 (0.68, 1.22); *P* = 0.52	1.02 (0.77, 1.34); *P* = 0.92	1.61 (1.10, 2.36); *P* = 0.02
	MMSE	0.88 (0.66, 1.17); *P* = 0.38	0.94 (0.70, 1.25); *P* = 0.66	0.72 (0.35, 1.49); *P* = 0.37
All Groups Combined				
	AQ	1.11 (1.03, 1.20); *P* = 0.008	1.08 (1.00, 1.16); *P* = 0.05	1.20 (1.09, 1.32); *P* <0.001
	FAQ	1.09 (1.01, 1.18); *P* = 0.03	1.16 (1.06, 1.26); *P* = 0.001	1.21 (1.11, 1.33); *P* <0.001
	MMSE	0.91 (0.81, 1.03); *P* = 0.15	0.84 (0.74, 0.95); *P* = 0.006	0.91 (0.74, 1.11); *P* = 0.35

Odds ratio (95% confidence interval); *P*-value; FDR significance level = 0.006; odds ratios indicate the association for a 1-point increase in AQ, FAQ, and MMSE change scores with FAST, GDS and CDR-GS score increase as the outcome. AD, Alzheimer’s disease; aMCI, amnestic mild cognitive impairment; AQ, Alzheimer’s Questionnaire; CI, confidence interval; CDR, Clinical Dementia Rating; CN, cognitively normal; FAQ, Functional Activities Questionnaire; FAST, Functional Assessment Staging Test; GDS, Global Deterioration Score; MMSE, Mini Mental State Exam; MoCA, Montreal Cognitive Assessment.

Correlation values for first and second year scores for each instrument are shown in Tables 5 and 6. The AQ and FAQ correlated strongly with FAST, GDS and CDR-GS values in both years while the MMSE correlated moderately with FAST, GDS and CDR-GS values in Year 1. In Year 2, the MMSE correlated moderately with the FAST and GDS, but demonstrated a strong correlation with the CDR-GS.

**Table 5:** **Correlation values for AQ, FAQ, MMSE, FAST, GDS and CDR Global Score for Year 1**

**Instrument**	**FAST Year 1**	**GDS Year 1**	**CDR-GS Year 1**
AQ Year 1	0.84	0.78	0.74
FAQ Year 1	0.83	0.77	0.75
MMSE Year 1	-0.59	-0.64	-0.65

*P* <0.001 for all correlations. AQ, Alzheimer’s Questionnaire; CDR, Clinical Dementia Rating; FAQ, Functional Activities Questionnaire; FAST, Functional Assessment Staging Test; GDS, Global Deterioration Scale; MMSE, Mini Mental State Exam.

**Table 6:** **Correlation values for AQ, FAQ, MMSE, FAST, GDS and CDR Global Score for Year 2**

**Instrument**	**FAST Year 2**	**GDS Year 2**	**CDR-GS Year 2**
AQ Year 2	0.80	0.80	0.75
FAQ Year 2	0.81	0.81	0.83
MMSE Year 2	-0.67	-0.72	-0.69

*P* <0.001 for all correlations. AQ, Alzheimer’s Questionnaire; CDR, Clinical Dementia Rating; FAQ, Functional Activities Questionnaire; FAST, Functional Assessment Staging Test; GDS, Global Deterioration Scale; MMSE, Mini Mental State Exam.

The mean change score for the AQ correlated weakly with the mean FAQ change score (r = 0.22, *P* = 0.002) while the MMSE mean change score demonstrated no correlation with the AQ mean change score (r = -0.02, *P* = 0.83).

## Discussion

Within the aMCI and AD groups the AQ demonstrated small sensitivity to change while its sensitivity to change in the CN group was trivial. In aMCI individuals the AQ, FAQ and MMSE all demonstrated small sensitivity to change. In the AD group, the MMSE and FAQ demonstrated greater sensitivity to change relative to the AQ. The AQ was also significantly associated with global change as measured by CDR-GS increase and correlated strongly with other established measures of global cognition and function. Although the effect sizes reported in this study are relatively small, they are consistent with the notion that cognitive changes associated with aMCI and AD are often subtle and difficult to detect from a psychometric standpoint. This point is a major challenge for researchers and clinical trialists as the variability of cognitive tests is often numerically similar to the rate of change [31]. Informant-based instruments that assess functional ability are also prone to high degrees of variability due to varying pre-morbid levels of function and gender differences in the degree of participation in many of the functional activities that are assessed [31]. The result is that when objective cognitive tests and informant-based instruments are used as endpoints in clinical trials the inherent variability of these measures often makes it difficult to detect true differences between placebo and treatment groups. However, others have suggested that lack of decline in placebo groups [32] and disease severity at baseline [31] can also significantly impact a trial’s ability to detect a significant treatment effect. The degree to which a particular cognitive or functional measure is responsive to changes in disease status is extremely important, particularly in pre-symptomatic and aMCI populations where cognitive decline is slower and more subtle [33].

The sample size calculations in the aMCI group demonstrate that the AQ is superior to the MMSE in terms of sensitivity to change; however, the AQ required a larger sample size than the FAQ. The sample size calculations highlight some important methodological issues in aMCI and AD studies that have been problematic. The first issue involves whether or not objective cognitive tests and informant-based instruments are sensitive enough to detect changes, particularly in earlier stages of aMCI and AD. Based on the results from the MMSE, our results suggest that the AQ may be superior to objective cognitive measures in detecting longitudinal change when compared on sample sizes required to detect a treatment effect. Although informant-based and objective cognitive assessments are often used in conjunction to assess drug efficacy, these results suggest that the MMSE is less sensitive to change over time than informant-based instruments.

Another issue these results highlight is that of instrument reliability as it relates to the required sample size needed to detect a treatment effect. There is a direct relationship between instrument reliability and sensitivity to change as instruments that are prone to higher variability between assessments may not detect significant longitudinal change as accurately as instruments with lower between-assessment variability. This imprecision ultimately leads to larger sample size requirements for clinical trials. Knopman and Caselli [34] point out that between-assessment variability is an inherent challenge when using patient-based objective cognitive tests to assess change, and longitudinal differences may be related to non-pathological factors, such as chance and regression toward the mean. Practice effects due to repeat administration of cognitive tests within relatively short periods of time also pose a significant threat to the ability to detect change associated with progression of aMCI/AD [35]. Others have also suggested that some objective cognitive tests are inherently insensitive to cognitive changes [36] and that variability between examiners using these instruments [37] is also a detrimental factor that prevents treatment effects from being observed. Although informant-based measures are more robust to some of these challenges than objective cognitive tests, they are still prone to some degree of measurement error, particularly in the area of inter-rater reliability [38].

In this study, the issue of reliability and its relationship to effect size was demonstrated in the AD group where the AQ yielded moderate sensitivity to change and the FAQ yielded large sensitivity to change. In this case, the effect size (corrected for reliability) for the FAQ was almost twice as large as that of the AQ. Some of this difference may be attributable to the higher reliability value of the FAQ which underscores the importance of not only an instrument’s psychometric ability to detect change, but also the ability of the examiner to administer the instrument in a way that can detect meaningful change. The importance of inter-rater reliability is highlighted by Kobak [39] who points out that reductions in inter-rater reliability, as measured by intra-class correlation, can result in significantly larger required sample sizes for clinical trials which stems from the increased measurement variability that reduces statistical power. This issue is also highlighted by Cummings *et al*. [40] who report that insufficient training and monitoring of examiners may lead to increased measurement variability which decreases the chance of detecting significant treatment effects. Connor and Sabbagh [41] also note that increases in measurement error may lead to decreases in instrument reliability, which results in a decreased ability to detect treatment effects.

The divergent sample size calculations for the AQ and FAQ may also be due to some of the inherent psychometric properties of each instrument. The FAQ captures not only the presence of impaired functioning, but also severity where the AQ only captures the presence of reported impairment in cognition and function. Thus, the inclusion of severity of impairment on the FAQ may account for the smaller required sample size calculation as a result of increased statistical power.

The results from the RCI calculations showed that the AQ identified clinically significant change in a larger percentage of individuals than did the FAQ and MMSE for aMCI individuals. The advantage that RCI scores provide is the ability to assess intra-individual change, which has been shown to have good predictive value in terms of cognitive decline [42]. The use of RCI scores in this context may provide a novel and more informative way to determine endpoints for aMCI and AD clinical trials. Since the majority of clinical trials for aMCI and AD rely on methods and analyses that simply assess group differences (for example, drug versus placebo) on a particular measure (for example, Alzheimer’s Disease Assessment Scale – cognition (ADAS-Cog)), it might be possible for drug efficacy to be assessed based on the percent of individuals showing clinically significant change on a measure, rather than just demonstrating a certain amount of change (for example, 25%) on an outcome measure.

One drawback to the current study is the relatively small sample size. Given that clinical trials often enroll hundreds of individuals, replication of these findings in a larger sample is needed in order to strengthen the argument for the AQ’s ability to detect longitudinal change. Autopsy confirmation of the clinical status for each individual would lend further support to the AQ’s ability to detect longitudinal change. Although the individuals participating in this study have agreed to an autopsy, many of them were still living at the time of the analysis so neuropathological confirmation of their clinical status was not available.

## Conclusions

The results of this study indicate that the AQ demonstrated small sensitivity to longitudinal cognitive changes associated with aMCI and AD. The AQ’s sensitivity to change in aMCI was comparable to the FAQ while both instruments outperformed the MMSE in terms of effect size and required sample size. The AQ was also significantly associated with longitudinal decreases in global cognition and function and was able to identify a greater proportion of aMCI individuals with clinically significant change when compared to other established measures. As clinicians and researchers continue to identify and treat individuals in earlier stages of AD, there is a need to utilize instruments that are sensitive to subtle cognitive changes over time. Although the AQ’s sensitivity to change was small, it is possible that its sensitivity to change may be enhanced when used in conjunction with sensitive objective cognitive tests and validated biomarkers of disease progression. In addition, the recent changes in mandatory screening measures for Medicare recipients as part of the Affordable Care Act may provide the opportunity for the AQ to be used by clinicians in order to satisfy the requirement for cognitive screening and might be helpful in detecting change over time in clinical settings.

## Abbreviations

AD: Alzheimer’s disease
aMCI: amnestic mild cognitive impairment
AQ: Alzheimer’s Questionnaire
CDR-GS: Clinical Dementia Rating Global Score
CN: cognitively normal
ES: effect size
FAQ: Functional Activities Questionnaire
FAST: Functional Assessment Staging Test
GDS: Global Deterioration Scale
MMSE: Mini Mental State Exam, Assessment
MoCA: Montreal Cognitive Assessment
RCI: reliable change index
